# Increase in lesion enhancement on gadoxetic acid enhanced MRI is associated with complete response to neoadjuvant chemotherapy in colorectal liver metastases

**DOI:** 10.1186/1470-7330-15-S1-P3

**Published:** 2015-10-02

**Authors:** S Islam, R Yin, A Riddell, H Tam, K Jhaveri, DM Koh

**Affiliations:** 1 Royal Marsden NHS Trust, Surrey, UK

## Aim

The aim was to determine whether the degree of enhancement on hepatocellular phase gadoxetic enhanced MRI and the apparent diffusion coefficient (ADC) before and after neoadjuvant chemotherapy could identify pathologic complete responders in colorectal liver metastases (CRLM).

## Methods

In this retrospective study, 22 patients (15 M: 7F ; mean age =65) with CRLM were evaluated with gadoxetic acid enhanced MRI before and after chemotherapy. Regions of interest were drawn encompassing metastases on T1W images and ADC map by an expert radiologist to record their average signal intensities (SI) normalised to the SI of paravertebral muscle and the average ADC value. We compared the median ADC value; pre-contrast and hepatocellular phase normalised SI and their percentage change in pathologic complete responders and pathologic non complete responders before and after chemotherapy using Mann-Whitney test. Receiver operating curve characteristics (ROC) of these parameters were determined. A p-value of < 0.05 was deemed statistically significant.

## Results

All patients received FOLFOX/FOLFIRI based-chemotherapy, while 8 received in addition bevacizumab. There were 37 CRLM at histology, of which 10 showed complete pathological response. There was a significant difference in the median percentage increase in the hepatocellular phase normalised SI of CRLM after neoadjuvant chemotherapy between pathologic complete responders (18%) and pathologic non complete responders (2.5%) (p = 0.04). By ROC analysis, an increase in the median hepatocellular phase normalised SI of 6% after chemotherapy has a sensitivity of 85% (95%CI:55-98%) and specificity of 70% (35-93%) for identifying pathologic complete responders (p=0.03). The other parameters including ADC were not statistically significant.

## Conclusions

An interval increase in the hepatocellular phase normalised SI of CRLM is associated with pathologic complete response following neoadjuvant chemotherapy.

